# Getting Everyone Onboard: Framing Collective Goal Progress Broadens Participation in Collective Marketing Campaigns

**DOI:** 10.3389/fpsyg.2019.02353

**Published:** 2019-10-18

**Authors:** Yaeeun Kim, Crystal Reeck

**Affiliations:** Department of Marketing and Supply Chain Management, Fox School of Business, Temple University, Philadelphia, PA, United States

**Keywords:** goal pursuit, interpersonal processes, social influence, fundraising, environmental decision making, political consumption

## Abstract

Collective marketing campaigns may feature goals that are not shared equally by all customers, such as a fundraiser for an environmental cause. For such campaigns, how can marketers encourage broad participation? The present research demonstrates that the framing of collective progress in such campaigns can broaden participation by highlighting the “large area” of progress toward the goal, emphasizing progress achieved for campaigns in their late stages and progress remaining in their early stages. We tested this large area hypothesis in the context of a waste reduction drive, examining the reactions of Democrats and Republicans who might be more or less inclined to support the drive respectively. Study 1 examined these processes when the drive was nearing completion, finding that an accumulating frame (focusing on progress achieved) increased motivation to participate for Republicans to levels comparable with Democrats. Study 2 evaluated these processes at earlier stages in the drive’s progress. In these circumstances, a remaining frame (focusing on contributions still needed) increased motivation to participate among Republicans to a similar level as Democrats. These findings indicate framings that highlight the large area in collective progress broaden participation in collective marketing campaigns, suggesting that marketers should highlight remaining contributions needed early on and accumulated contributions received later in collective marketing campaigns.

## Introduction

When conducting a collective campaign such as a fundraiser or a donation drive, campaigners often create a graphical measure, like a thermometer, to publicly track contributions. The first thermometer to be used as a campaign tracker was for a 1905 YMCA fundraiser that raised $4 million in less than 1 month (Cutlip, 1965). Similar or identical graphical trackers are ubiquitous in contemporary marketing campaigns and have been used on crowdfunding sites as well as when gathering signatures for a petition.

While this approach is effective when the goals are shared among all participants, it remains unclear whether the same is true when participants are less likely to share a collective goal equally. For example, fundraising for the local police department might face skepticism from the Black Lives Matter community and a campaign to reduce a business’s carbon footprint might meet resistance from climate change skeptics. Previous research has investigated how information about progress toward achieving a goal can motivate behavior in individual goal pursuit (Koo and Fishbach, 2008; Robinson et al., 2012; Huang et al., 2014) or collective goal pursuit (Fishbach et al., 2011; Cryder et al., 2013; Fishbach and Tu, 2016). Recent literature showed that prosocial choice is driven by a generalized morality preference for doing the right thing (Capraro and Rand, 2018; Tappin and Capraro, 2018), which is effective when it is perceived as a norm (Capraro et al., 2019) and framed socially (Capraro and Vanzo, 2019). Other psychological processes likely shape goal pursuit when not everyone is committed to the collective goal, and in such circumstances, there may be more effective methods of promoting participation. Furthermore, does one’s reaction toward others’ contribution change in the early versus late stages of progress? How do others’ actions influence individuals’ participation toward a collective goal?

To address this issue, the current paper investigated how the presentation of campaign progress affects broad participation in a collective marketing campaign when a subset of consumers are less likely to support the goal. In recent years, Americans have become more polarized based on political identity, with ramifications for a range of consumer preferences including brand preferences (Khan et al., 2013), varied tastes (Carney et al., 2008), in-group conformity, and sustainable consumption behaviors (Kidwell et al., 2013). The research context of our paper was an environmentally-friendly waste reduction drive where some people are more supportive and some people are less supportive to the goal. We analyzed participation based on political party affiliation. We expected that Democratic participants would be more likely to share the drive’s goal and thus be more motivated to donate, while Republican participants would be less likely to share the drive’s goal and thus less motivated to donate.

Findings revealed that highlighting the larger area of the progress bar tracking collective contributions had the effect of broadening participation in the campaign by motivating Republicans to levels of participation similar to Democrats, who despite not necessarily sharing the drive’s goal remained sensitive to the framing of collective progress. This broader participation arises due to goal desirability, perceptions of the impact of one’s participation, and desire to help the community. These findings enrich theories of goal pursuit in the social domain and indicate that marketers can broaden participation in collective marketing campaigns by highlighting the larger area of progress bars used to track contributions.

We first provide a theoretical background of the psychological processes underlying goal pursuit and articulate our hypotheses regarding collective goals that are not shared by all consumers. We then report the results from two experiments testing these hypotheses and discuss the theoretical contributions of this work as well as the managerial implications.

## Literature Review and Hypotheses

### Goal Pursuit

Prior literature has highlighted two fundamental components of goal pursuit: goal commitment and goal progress (Fishbach and Dhar, 2005; Koo and Fishbach, 2008; Bonezzi et al., 2011; Cryder et al., 2013). Goal commitment refers to a person’s attachment to or determination to reach a goal (Hollenbeck and Klein, 1987; Locke et al., 1988; Brunstein, 1993; Velasco Moreno et al., 2019). Empirical studies demonstrate that commitment increases motivation (Koo and Fishbach, 2008), promoting goal-consistent actions early in goal-pursuit (Fishbach and Dhar, 2005; Zhang and Huang, 2010). However, it remains unclear whether one’s motivation toward pursuing a collective goal with others can be increased if the goal is not equally shared by everyone.

In contrast, goal progress refers to the concrete progress made toward achieving a goal (Koo and Fishbach, 2008). Typically, people’s efforts toward goal pursuit increase with proximity to achieving the goal (Hull, 1932; Kivetz et al., 2006), referred to as the goal-gradient effect. For example, in the context of customer loyalty programs, customers make more purchases as they approach loyalty rewards goals, and this increased motivation is linked to perceptions of the perceived impact of each action (Kivetz et al., 2006). In the context of collective goals, people are more likely to participate in the later stages of progress (Cryder et al., 2013). However, it is unclear whether similar processes shape motivation when collective goals are not shared by all.

### Progress Framing

Research in judgment and decision making shows that perceptions are sensitive to relative comparisons (Kahneman and Frederick, 2002). By highlighting relative actions, goal progress can be framed as to-date (i.e., accumulating) or to-go (i.e., remaining, Koo and Fishbach, 2008). In individual goal pursuit, the accumulating goal progress frame is typically more motivating than the remaining progress frame when the goal progress is low while the remaining frame is typically more motivating than the accumulating frame when goal progress is high (Koo and Fishbach, 2012). Essentially, if portraying progress graphically, one would prefer to highlight the small area of the progress bar in individual goal pursuit (emphasizing progress achieved early and progress remaining later in goal pursuit). There is some evidence, however, that different processes may shape behavior with collective goal pursuit. Prior work has shown that people who are less likely to respond to a fundraising request due to lack of prior participation are more motivated to participate in later stages of fundraising when accumulated progress is emphasized (Koo and Fishbach, 2008). The present work builds on this initial finding by examining controversial goals where some participants may be ambivalent about or antagonistic toward the collective goal. Furthermore, we examine what underlying processes may guide motivation to participate in this context. Building on these earlier findings, we anticipate that highlighting the larger area of collective goal progress may broaden overall participation when social goals are not universal. Near the completion of the drive, underscoring accumulated progress serves as a signal of others’ commitment, evoking social norms to encourage participation by additional consumers (Goldstein et al., 2008) and supplementing the commitment of those who might not otherwise share the goal. By highlighting others’ actions, emphasizing progress-to-date also serves as a reminder of what others are likely to do. With prosocial campaigns and collective endeavors, this intervention may share roots with moral nudges, which have been shown to motivate prosocial behavior (Tappin and Capraro, 2018; Capraro and Vanzo, 2019) and increase charitable donations (Capraro et al., 2019) and decrease tax evasion (Bott et al., 2017). However, highlighting the remaining progress signals a low need for progress, which may not spur engagement. At the same time, we anticipate that there will be a ceiling effect for those who are already motivated to participate in the collective drive, and that progress framing will thus have a minimal effect. We therefore predict that:

H1 Those who share the collective campaign’s goal will be motivated to participate in the drive regardless of progress framing.
H2 In the end stages of collective campaigns, highlighting the large area (accumulated progress) will increase motivation to participate among those who are less likely to share the campaign’s goal to levels similar to those who are more likely to share the campaign’s goal.

On the other hand, at the beginning stages of a collective campaign, when overall motivation is often low (Koo and Fishbach, 2012), we predict that the processes underlying motivation to participate will differ. Contrary to the positive impact that the accumulating frame has on motivation when progress is high during the later stages of a campaign, the accumulating frame in the early stage can suggest low commitment made by others thus far. Highlighting progress to date can therefore be demotivating when collective progress is low during the early stages of a campaign, particularly for those who are less likely to share the goal of the drive. Instead, we predict that the remaining frame would increase motivation among those who are less likely to share the goal by signaling the need for progress. As a result, a large area in the progress bar may broaden overall participation when social goals are not universal. Thus, we expect that:

H3 During the early stages of a collective campaign, the large area (remaining frame) will increase motivation to participate among those who are less likely to share the goal to levels similar to those who are more likely to share the campaign’s goal.

### Psychological Processes and Collective Goal Pursuits

As outlined earlier, we posit that three psychological processes underlie the broadened participation observed when an accumulating progress frame is employed near goal completion: perceived goal desirability, perceived impact of participation, and feelings of helping the community. First, observing others’ high overall participation leads to judgments that the goal is desirable to pursue. Desirability refers to the value of the end state of an action (Liu, 2008). Particularly when pursuing goals with others, high levels of participation by others signal a social norm that this action is desirable, thus encouraging others to also pursue the goal (Goldstein et al., 2008). In effect, observing others’ participation signals a social goal commitment, which can supplement one’s own low individual commitment to the drive’s goal and spur participation. Thus, we anticipate:

H4 As a collective campaign nears completion, the accumulating frame motivates participation among those who are less likely to share a collective goal by enhancing the perceived desirability of the drive’s goal.

Second, perceptions of the impact of one’s actions also likely increase motivation to participate in collective campaigns as they near completion. The perceived impact of one’s participation increases as greater progress is made toward achieving the goal (Cryder et al., 2013), and people are more likely to contribute to a collective goal when their actions have a greater impact (Sen et al., 2001; Kivetz et al., 2006; Fishbach and Tu, 2016). We therefore hypothesize:

H5 As a collective campaign nears completion, the accumulating frame motivates participation among those who are less likely to share a collective goal by enhancing their perceptions of the impact of their participation.

Third, increasing participation by others in a collective campaign signals that the goal is shared by the general community, thus increasing perceptions that one’s participation would contribute to the community. Research across multiple domains of consumption (e.g., Crane, 2001; Mazar and Zhong, 2010; Olson et al., 2016) suggest that such feelings of helping can spur diverse prosocial behavior. These feelings of helping the community increase moral obligation, in turn promoting prosocial behaviors (De Groot and Steg, 2009). Thus, we propose:

H6 As a collective campaign nears completion, the accumulating frame motivates participation among those who are less likely to share a collective goal by enhancing their feelings of helping the community.

We conducted two studies to test our hypotheses in the context of an environmentally-friendly waste reduction drive. Consumer attitudes toward pro-environmental initiatives have been found to vary based on political party affiliation (Jost et al., 2003; Dunlap and McCright, 2008; Feygina et al., 2010; Kidwell et al., 2013), so given the marketing and policy ramifications of political attitudes (Ordabayeva and Fernandes, 2018), we used political party affiliation as a proxy of different levels of support toward environmentally-friendly campaigns. By using political party affiliation to operationalize support for the collective goal, this research approach avoided potential issues arising from attempting to measure participants’ support for the goal in advance, such as potentially priming responses or evoking experimenter demand. Moreover, as political polarization among consumers in the United States continues to grow (Haidt and Graham, 2007; Winterich et al., 2012; Talhelm et al., 2015), it is important to understand how such divides shape consumer behavior.

Study 1 examined how progress framing alters motivation to participate as the drive nears completion, while Study 2 examined the drive in its early stages. Taken together, these experiments investigate how framing collective progress can broaden participation in such drives, examining the psychological processes that shape willingness to participate among those who are more or less likely to support the goals of a collective marketing campaign.

## Study 1: Collective Goal Near Completion

Study 1 examined people’s motivation to participate in a waste reduction drive as it neared completion. This campaign used collective progress to highlight either accumulating progress achieved or remaining progress necessary. While we anticipated Democrats’ motivation would be high regardless of framing given the alignment between party environmental attitudes and the waste reduction drive, we expected Republicans’ motivation would depend upon the framing of collective progress, with accumulating frames promoting participation.

### Study Design

**FIGURE 1:**
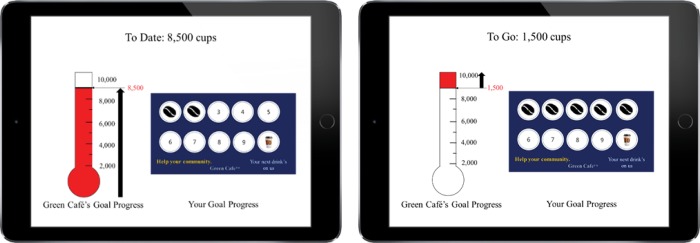
Scenario (Studies 1 and 2) and examples of stimuli for Study 1. Participants were informed of the waste reduction drive, with the thermometer tracking collective progress. The accumulating frame and two-stamp condition is shown on the **left** while the remaining-frame and five-stamp condition is shown on the **right**.

Three hundred seventy-nine participants (183 female, *M*_age_ = 36.1, *SD* = 18.8) were recruited from Amazon Mechanical Turk for an online experiment. Participants were based in the United States with an approval rate of at least 97% on Amazon Mechanical Turk having completed at least 100 HITs. Participants were instructed to imagine they received an incomplete loyalty card for a local café from a friend who was moving away from the area (revised from Koo and Fishbach, 2012). The loyalty card included information about an environmentally-friendly waste reduction drive the café was conducting, with the goal of saving 10,000 disposable cups from the landfill by asking customers to bring their own reusable mugs (see Figure 1). Each purchase using one’s own mug instead of using a disposable coffee cup saved one cup from the landfill and earned a stamp on the loyalty card. A completed reward card (10 stamps) could be exchanged for a free beverage at the café. The waste reduction drive’s progress was monitored using a thermometer displayed on the point-of-sale device.

Study 1 tested a 2 (individual progress: two-stamp vs. five-stamp) × 2 (frame of collective progress: accumulating vs. remaining) × 2 (political affiliation: Republican vs. Democrat) interaction effect on the motivation of participation in a collective goal. While the overall amount of collective progress was identical in all conditions, the framing of collective progress was manipulated to highlight either accumulating progress (i.e., 8,500 cups saved to-date) or remaining progress (i.e., 1,500 cups to-go). To ensure that effects of collective progress framing generalized across different levels of individual progress, we also manipulated the number of existing stamps on the loyalty card (2 or 5 stamps for low or high progress, respectively). We asked an attention check question on a seven-point scale, “If you are paying attention to this survey, select the second button.” It was a single question and participants were not forced to respond. There is a one seventh (14.3%) chance that a participant inadvertently passes the question. Forty-eight participants answered this question incorrectly and were removed from all analyses. To avoid priming identities, political party affiliation was measured at the end of the survey. As political party affiliation was used to infer predispositions toward the environmentally-friendly goal, data were analyzed only from participants who identified as Republican or Democrat (*n* = 264, 80 Republicans, 184 Democrats).

After reviewing the loyalty card, participants were asked to answer several questions on seven-point scales. They rated how likely they would be to use the reward card (i.e., “How likely is it that you would visit Green Café and use the reward card?” 1 = Definitely would not, 7 = Definitely would) and the extent to which they felt Green Café’s goal to decrease waste was desirable (i.e., “Is Green Café’s goal to decrease waste desirable?” 1 = Strongly disagree, 7 = Strongly agree). Participants also responded to a six-item scale survey regarding the perceived impact of their participation by evaluating the extent to which getting one stamp makes progress toward reducing waste (e.g., “My participation will have a significant effect on the likelihood of a successful drive” 1 = Strongly disagree to 7 = Strongly agree). They also rated how much they felt participating would help their community (i.e., “To what extent does getting one stamp make you feel that you are helping your community?” 1 = Not at all, 7 = Very much). To confirm that the framing of collective progress did not change perceptions that the waste reduction drive would be successful, participants completed a three-item scale of the campaign’s likelihood of success (e.g., “To what extent do you trust Green Café to efficiently reduce its waste?” 1 = Not at all, 7 = Very much; *a* = 0.76; see Supplementary Web Appendix).

### Results and Discussion

**FIGURE 2:**
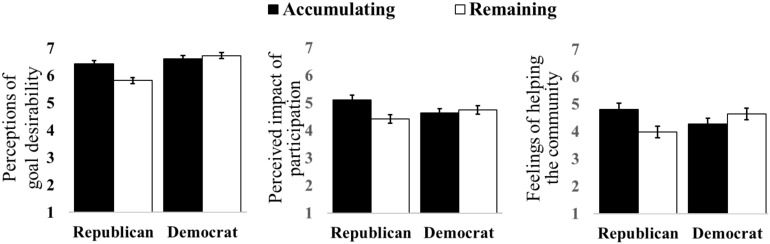
Study 1 Results: Collective progress framing by political affiliation for perceptions of goal desirability **(left)**, perceived impact of participation **(center)**, and feelings of helping the community **(right)**.

Ratings of the desirability of the goal of the waste reduction drive were analyzed using an ANOVA with the between-subjects factors of collective progress framing (accumulating or remaining), individual progress (2 or 5 stamps), and political affiliation (Republican or Democrat). Democrats rated the goal as more desirable (*M* = 6.7) than Republicans [*M* = 6.1, *F*(1, 256) = 19.86, *p* < 0.001], as expected. The interaction between individual progress and political affiliation was also significant [*F*(1, 256) = 3.88, *p* = 0.050]. Additionally, we found that there was a larger difference between Democrats and Republicans when the loyalty card already had two stamps [*M*_Dem_ = 6.7, SD = 0.59 vs. *M*_Rep_ = 5.8, *SD* = 1.82; Welch’s *t*(34.3) = 2.87, *p* = 0.007] than when it had five stamps [*M*_Dem_ = 6.6, *SD* = 0.62 vs. *M*_Rep_ = 6.3, *SD* = 1.16; Welch’s *t*(61) = 1.76, *p* = 0.083]. Importantly, the analyses yielded the predicted interaction between progress framing and political affiliation [*F*(1, 256) = 7.67, *p* = 0.006; see Figure 2]. As expected, in the remaining frame condition, Democrats rated the café’s waste reduction goal as more desirable (*M* = 6.7, *SD* = 0.52) than Republicans [*M* = 5.8, *SD* = 0.68; Welch’s *t*(46.9) = 3.54, *p* = 0.001]. This is the typically expected pattern, as Democrats are generally more supportive of environmentally-friendly efforts than Republicans. However, in the accumulating frame condition, both Democrats and Republicans rated the goal as similarly desirable [*M*_Dem_ = 6.6, *SD* = 0.68 vs. *M*_Rep_ = 6.4, *SD* = 1.16; Welch’s *t*(45.1) = 0.95, *p* = 0.349], supporting Hypothesis 2. The accumulating frame enhanced Republicans’ evaluation of the desirability of waste reduction, causing them to perceive the waste reduction goal as favorably as Democrats did. No other effects or interactions were significant.

Building on these findings, we next examined the extent to which participating in the drive made one feel as if they were contributing to the drive’s success. Ratings of the perceived impact of participation were submitted to the same between-subjects ANOVA used to examine effects on perceptions of the drive’s desirability. Supporting Hypotheses 1 and 2, the results revealed a significant interaction between progress framing and political affiliation [*F*(1, 256) = 5.38, *p* = 0.021; see Figure 2]. Democrats rated the perceived impact of their participation similarly regardless of frame [*M*_*accumulating*_ = 4.6, *SD* = 1.14 vs. *M*_*remaining*_ = 4.7, *SD* = 1.28; *t*(182) = 0.67, *p* = 0.501], whereas Republicans rated the perceived impact greater in the accumulating frame (*M* = 5.1, *SD* = 1.13) compared to the remaining frame [*M* = 4.4, *SD* = 1.53; Welch’s *t*(77.3) = 2.26, *p* = 0.027]. No other effects or interactions were significant. These results indicate that the accumulating frame is more effective at enhancing Republicans’ judgments of the impact of their participation, consistent with our hypotheses.

Third, we conducted a similar analysis examining participants’ ratings of the extent to which participating made them feel they were helping their community. In alignment with our other analyses, the interaction between collective progress framing and political affiliation was significant [*F*(1, 256) = 6.75, *p* = 0.010; see Figure 2]. Democrats’ feelings of helping the community did not significantly vary with collective progress framing [*M*_*accumulating*_ = 4.3, *SD* = 1.58 vs. *M*_*remaining*_ = 4.6, *SD* = 1.67; *t*(182) = 1.51, *p* = 0.134], while Republicans reported marginally greater feelings of helping in the accumulating frame (*M* = 4.8, *SD* = 1.70) than in the remaining frame [*M* = 4.0, *SD* = 2.09; *t*(78) = 1.92, *p* = 0.059]. No other effects or interactions were significant. These findings are consistent with our hypotheses that the accumulating frame would boost judgments regarding the collective appeal of the waste reduction drive and engender a social norm to participate, therein enhancing Republicans’ judgments of the drive.

Our initial analyses revealed that the accumulating frame of collective progress enhanced Republicans’ perceptions of the goals of the drive, their judgment of the impact of their participation, and their feelings that participating would help their community. However, it is possible that the accumulating frame changed perceptions that the drive would be successful. To address this potential explanation for the results, we analyzed ratings of the likelihood that the waste reduction drive would be successful. Importantly, the drive’s perceived success did not differ based on the framing of collective progress [*M*_*accumulating*_ = 5.6 vs. *M*_*remaining*_ = 5.5, *F*(1, 262) = 0.35, *p* = 0.554], consistent with previous research (Koo and Fishbach, 2012). These results indicate differences in the perceived success of the drive cannot account for the key findings observed here.

We next sought to test whether perceptions of the desirability of the drive’s goal, judgments of the impact of participation, and feelings of helping the community mediate the effect of collective framing progress on participation in the drive for Republicans (Hayes, 2013, model 7). A moderated mediation model revealed that Republicans decrease their willingness to use a reward card in the remaining compared to the accumulating frame (95% CI: −0.66 and −0.02) and that this decrease is mediated by changes in perceived desirability of the drive’s goal, while this effect is not observed for Democrats (95% CI: −0.03 and 0.22), supporting hypothesis 4 (see Table 1). As the direct effect of framing on willingness to use the reward card was not significant (β = −0.02, *p* = 0.923), this finding indicates an indirect-only mediation (Zhao et al., 2010). In short, the accumulating frame increases the willingness to use the reward card by increasing the perceived desirability of the goal. Similar results were found in models examining the perceived impact of participation (Republican 95% CI: −0.86 and −0.07; Democrat 95% CI: −0.16 and 0.30) and feelings of helping the community (Republican 95% CI: −0.76 and −0.01; Democrat 95% CI: −0.04 and 0.37). These findings support Hypotheses 5 and 6 respectively (see Supplementary Web Appendix).

**TABLE 1:** Perceptions of goal desirability mediate the effects of progress framing on intention to participate in the waste reduction drive for Republicans, but not for Democrats.

	**Consequent**							
		***M* (Perceptions of goal desirability)**				***Y* (Willingness to use a reward card)**		
**Antecedent**		**Coeff.**	***SE***	***p***		**Coeff.**	***SE***	***p***
*X* (Progress framing)	*a*_1_	−1.309	0.446	0.004	*c*′*_1_*	−0.017	0.177	0.923
*M*		–	–	–	*b*	0.578	0.089	<0.001
*W*(Political affiliation)	*a*_2_	0.194	0.186	0.297	*c*′_2_			
*X × W*	*a*_3_	0.711	0.254	0.006				
Constant	*i*_*M*_	6.222	0.330	<0.001	*i*_*Y*_	1.878	0.600	0.002
		*R*^2^ = 0.103				*R*^2^ = 0.139		
		*F*(3, 260) = 9.918, *p* < 0.001				*F*(2, 261) = 21.034, *p* < 0.001		

By entering all of the three measures as simultaneous mediators in the moderated mediation model, we found that the perceived desirability of the goal (index: 0.24, 95% CI: 0.01 and 0.48) and the perceived impact of participation (index: 0.38, 95% CI: 0.05 and 0.79) mediate the effect of progress framing on participation. The 95% CI obtained for the index of moderated mediation of feelings of helping (−0.11 and 0.33) included zero, which suggests that the indirect effect was absent when the three mediators were tested together. Thus, Republican participants were more likely to participate in the accumulating frame due to increased perceived desirability of the drive’s goal and a higher perceived impact of their participation.

## Study 2: Collective Goal at Earlier Stages

In Study 1, we found that the accumulating frame of collective progress increases Republicans’ motivation to participate by enhancing the desirability of the goal, the perceived impact of participation, and feelings of helping the community. However, it remains unclear whether similar effects would emerge for collective goals at earlier stages, with lower amounts of accumulated progress. This is particularly important from a managerial perspective, as marketers often desire to boost participation for new campaigns. Therefore, Study 2 tests Hypothesis 3 by examining how progress framing shapes participation and perceptions of the brand conducting the drive when the waste reduction drive is at earlier stages of progress.

### Study Design

Three hundred eighty-five US participants (176 female, *M*_age_ = 37.0, *SD* = 12.1) were recruited through Amazon Mechanical Turk and randomly assigned to one of four conditions in a 2 (frame of collective progress: accumulating or remaining) × 2 (progress of collective goal: 1,500 cups or 5,000 cups) design. As individual progress did not interact with our key findings from Study 1, in this experiment, we did not manipulate individual progress and all participants viewed a reward card with three stamps. Participants were based in the United States with an approval rate of at least 97% on Amazon Mechanical Turk having completed at least 100 HITs, and participants who had completed our previous study were excluded from participation. We asked participants to answer the same attention check question used in Study 1. Fifty-nine participants who did not pass the attention check were removed from all analyses.

Participants followed the same procedure of Study 1. After learning about the waste reduction drive, participants rated their motivation to participate in the drive, how long it would take them to complete the reward card, brand liking (e.g., “I like the brand Green Café,” 1 = Strongly disagree, 7 = Strongly agree; *a* = 0.92), and feelings of helping the environment (i.e., “To what extent does getting one stamp make you feel that you are helping the environment?” 1 = Not at all, 7 = Very much). As in Study 1, participants completed a three-item scale of the campaign’s likelihood of success (*a* = 0.80; see Supplementary Web Appendix). At the end of the survey, participants reported their political affiliation and only responses from Republicans or Democrats were analyzed (*n* = 257, 86 Republicans, 171 Democrats).

### Results and Discussion

Ratings of the intention to participate in the drive and bring one’s own mug to the café were analyzed using an ANOVA with the between-subjects factors of collective progress framing (accumulating or remaining), drive stage (1,500 or 5,000 cups saved), and political affiliation (Republican or Democrat). Analyses revealed a marginally significant interaction between framing and political affiliation on one’s motivation to participate in the drive [*F*(1, 249) = 3.55, *p* = 0.061]. As expected, Democrats’ intentions to participate (*M* = 6.3, *SD* = 1.01) were higher than Republicans’ in the accumulating frame [*M* = 5.6, *SD* = 1.67; Welch’s *t*(66) = 2.54, *p* = 0.013] but did not differ in the remaining frame [*M*_Dem_ = 6.0, *SD* = 1.50 vs. *M*_Rep_ = 6.0, *SD* = 1.61; *t*(136) = 0.02, *p* = 0.987], supporting hypothesis 3. No other main effects or interactions were significant. In sum, when overall participation is low, Democrats remain consistently motivated to participate in the goal. However, the remaining frame promoted Republicans’ willingness to participate in the campaign, such that their indicated willingness to participate did not differ from Democrats’.

**FIGURE 3:**
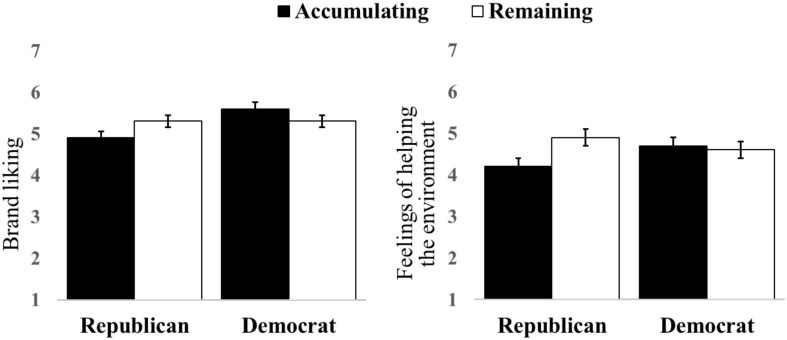
Study 2 Results: Collective progress framing by political affiliation for brand liking **(left)** and feelings of helping the environment **(right)**.

We next examined ratings of brand liking using a similar ANOVA. There was a marginally significant main effect of political affiliation, as Democrats evaluated the brand more favorably (*M* = 5.4) than Republicans [*M* = 5.1, *F*(1, 249) = 3.63, *p* = 0.058]. The interaction between collective progress framing and political affiliation was significant [*F*(1, 249) = 4.66, *p* = 0.032; see Figure 3]. As expected, Democrats evaluated the brand more favorably (*M* = 5.6, *SD* = 1.03) than Republicans in the accumulating frame [*M* = 4.9, *SD* = 1.36; Welch’s *t*(76.9) = 2.87, *p* = 0.005]. However, in the remaining frame, participants rated the brand favorably regardless of political affiliation [*M*_Dem_ = 5.3, *SD* = 1.31 vs. *M*_Rep_ = 5.2, *SD* = 1.41; *t*(136) = 0.30, *p* = 0.767]. No other main effects or interactions were significant. The interaction reveals attitudes toward the brand are favorable in the remaining frame across political affiliations, whereas only Democrats liked the brand in the accumulating frame. Similar results were found examining feelings of helping the environment using the same ANOVA approach. There was a marginally significant interaction between collective progress frame and political affiliation [*F*(1, 249) = 3.65, *p* = 0.057; see Figure 3]. These findings are consistent with our hypotheses that the remaining frame would boost evaluation regarding the collective appeal of the waste reduction drive and engender social norms to participate, therein enhancing Republicans’ evaluation of the drive. Importantly, as with Study 1, these findings were not driven by differences in judgments that the drive would be successful, as ratings regarding the drive’s likely success again did not differ based on progress framing [*M*_*accumulating*_ = 5.3 vs. *M*_*remaining*_ = 5.4, *F*(1, 255) = 0.13, *p* = 0.714]. In sum, the remaining frame increased Republicans’ feelings of helping the environment, whereas the goal framing did not change Democrats’ feelings.

To summarize, findings from Study 2 support the large area hypothesis. When highlighting the remaining progress needed, which is a larger area in the progress bar, Republicans rated the brand as favorably as Democrats and reported similar feelings of helping the environment if they participated. Overall, the remaining frame was most effective for broadening support for these earlier stage drives.

## General Discussion

The present research examines how framing collective campaign progress affects participation in a marketing campaign. In the context of a waste reduction drive, we find that Democrats’ interest in participating is generally high regardless of how collective progress toward achieving the goal is framed. However, Republicans are sensitive to collective progress framing. Overall, framing feedback about collective goal progress to signal information of the large contributions made by others at the end stage and the large need for progress at the beginning stage of a campaign served to broaden participation. Near the end of the campaign, this large area effect was mediated by changes in Republican participants’ perceptions of goal desirability, the impact of their participation, and feelings of helping.

The current research expands understanding of the social processes influencing collective goal pursuit. Previous studies have typically focused on non-controversial social goals, such as donations to charities that most consumers would support (Koo and Fishbach, 2008; Bonezzi et al., 2011; Fishbach et al., 2011, 2016; Cryder et al., 2013; Fishbach and Tu, 2016), while the present studies instead examine a collective goal that is not shared by all consumers. To be clear, this paper investigates goal pursuit in a social context. Consistent with previous literature, the current research demonstrates that perceiving the goal as desirable (Koo and Fishbach, 2008) and one’s contribution as impactful (Fishbach et al., 2011; Cryder et al., 2013; Velasco Moreno et al., 2019) spurs participation. However, the present work revealed that these processes are influenced by collective progress framing for those who might be disinclined toward the goal. In contrast to studies focused on individual goal pursuit (Koo and Fishbach, 2012), the present studies of collective goal pursuit demonstrated that a remaining progress frame was most effective early in goal pursuit while an accumulating progress frame was most effective late in goal pursuit. These divergent findings may arise from differences between individual and collective goal pursuit. For example, in collective goal pursuit, social considerations may play a larger role in shaping motivations. Considerations of the needs of others or the likely motivation for others’ actions may become more salient by highlighting the great need for progress early on and a high amount of achieved progress later on, thus spurring people to participate. Consistent with this possibility, increased perceptions of the goal’s desirability likely arise due to inferences drawn from observing others’ high contributions and mediate the effects observed in Study 1, and feelings of helping appear to track responses to framing in both studies. These processes are less likely to play a role in shaping individual goal pursuit, as such social considerations are more limited (Fishbach and Dhar, 2005; Novemsky and Dhar, 2005; Schumpe et al., 2018). The role of goal desirability in shaping motivation here is consistent with theories of goal commitment that emphasize the role of both the expectation that the goal will be achieved and the value of pursuing the goal (e.g., Liberman and Förster, 2008; Zhang and Huang, 2010). The current findings indicate that the accumulating progress frame helps supplement the goal commitment of those who are disinclined toward the goal by increasing the perceived desirability of the goal.

The present findings also contribute to a growing literature examining how political identities shape consumer behavior. In the present research, we used political affiliation as a proxy for likely support of the goal of the waste reduction drive, a context in which support is broad among liberal consumers and likely more variable among conservative consumers. One interesting avenue for future research is to examine whether the present findings are shaped by cognitive and attitudinal differences between liberals and conservatives, such as sensitivity to different appeals (Winterich et al., 2012; Kidwell et al., 2013), adherence to different moral values (Haidt and Graham, 2007; Graham et al., 2009, 2011), and diverse desires for differentiation (Ordabayeva and Fernandes, 2018). Several possibilities arise regarding whether the findings related to broadening the participation of conservative consumers generalize to liberals. One possibility is that these results generalize to other groups that are less likely to support a campaign’s goal, such as Black Lives Matter activists responding to a fundraiser for the police department. Indeed, some prior research suggests that even stronger effects might emerge with liberal consumers, who have shown greater effects of political ideology on consumption than conservative consumers (Jost et al., 2017; Jung et al., 2017).

Another possibility is that effects of framing on participation may be similar for both liberals and conservatives, but may be driven by different underlying psychological processes. For example, conservative consumers may be more likely to consider the reaction of an in-group to their behavior, while liberals may be more likely to consider the fairness of their actions to others (Jost et al., 2017). Interrogating these possibilities make for a rich area for future research that would inform work on both political consumption and social goal pursuit. Relatedly, our finding that highlight accumulating progress results in similar motivation to participate for both Democrats and Republicans in the current study could be the results of a “moral nudge.” Highlighting what others are likely to do has been shown to increase prosocial behavior in economic games (Tappin and Capraro, 2018; Capraro and Vanzo, 2019), spur charitable giving (Capraro et al., 2019), and decrease tax evasion (Bott et al., 2017). Both the progress manipulation employed here and moral nudges share the feature that they draw attention to what others do. Here, we also demonstrate that highlighting accumulating progress is late stage campaigns operates by signaling that the goal is valuable, in addition to helping one’s community.

One interesting aspect of the individual goal pursuit literature is the finding that motivation may be at its lowest in the middle of goal progress (Bonezzi et al., 2011; Huang, 2018). This effect may emerge because new progress achieved may appear marginal in contrast to both previous progress achieved and progress remaining. We don’t find any differences between early-stage and middle-stage collective drive progresses in the present research, suggesting this effect may be governed by different mechanisms in the context of collective goal pursuit. However, more research should examine this question. In the present studies, motivation to participate was at ceiling for Democrat participants. As a result, they exhibited less sensitivity in the current studies to the processes that were found to guide Republican participants’ decisions to participate. Future studies featuring less polarizing goals might avoid such ceiling effects and potentially allow examination of these processes in both groups.

The present findings have important practical implications for brands orchestrating collective marketing campaigns. First, we demonstrate that the framing of collective progress broadens participation in collective marketing campaigns. Organizations seeking to spur participation in such campaigns should thus highlight remaining contributions needed early in the campaign and accumulated contributions received later in the campaign. Second, these findings are timely, given the growing trend for brands to incorporate more political considerations into their advertising (Kim et al., 2018). The current results suggest that the framing of information may be used to avoid alienating consumers that are less likely to support the political attitudes incorporated into the advertising campaigns. Finally, these results broadly address customer relationship management, as the context for the waste reduction drive was a customer loyalty program and the framing had ramifications for brand perceptions. As political identities begin playing a larger role in consumer behavior, identifying means such as those studied here to appeal to all consumers can boost loyalty and avoid the potential for brand relationships to conflict with political views.

The present research does, however, have a few limitations. First, the campaign scenarios employed here are hypothetical. We used this approach to avoid associations with actual brands or stores which might confound the findings, but future work should examine these processes in an incentive-compatible design. Second, it would be ideal if we had examined both early and late stage campaigns within the same experiment to allow for more direct comparison of the underlying psychological mechanisms. Third, while we used political party affiliation as a proxy for likely support of the campaign goal, it would be helpful if future research employed a more direct index of participants’ initial commitment to the communal goal. Finally, the campaign employed in the present research included a direct benefit to those who chose to participate in the campaign, as they moved closer to earning a free beverage. Future research could examine more altruistic campaigns, such as charitable donations, where participation does not produce a tangible direct benefit.

## Conclusion

In conclusion, the present experiments demonstrate that framing information regarding others’ actions shapes motivation to participate in a collective marketing campaign. Highlighting remaining progress needed earlier in a campaign and accumulated progress achieved later in a campaign broadens its appeal to consumers who might otherwise have been unlikely to share the campaign’s goal. Perceptions of goal desirability, the impact of one’s contributions, and feelings of helping appear to underlie this broadened motivation. These findings can inform both marketers and policymakers seeking to conduct successful collective campaigns.

## Data Availability Statement

The datasets for this study can be found in the Open Science Framework: http://osf.io/akm8g/.

## Ethics Statement

The studies involving human participants were reviewed and approved by the Temple University Institutional Review Board. Written informed consent for participation was not required for this study in accordance with the national legislation and the institutional requirements.

## Author Contributions

YK and CR jointly designed, analyzed, wrote up this manuscript, and undertook all data collection.

## Conflict of Interest

The authors declare that the research was conducted in the absence of any commercial or financial relationships that could be construed as a potential conflict of interest.
