# Supplementary material for: Getting Everyone Onboard: Framing Collective Goal Progress Broadens Participation in Collective Marketing Campaigns
Source: Front Psychol. 2019 Oct 18;10:2353. doi: 10.3389/fpsyg.2019.02353 (PMC6813241; doi:10.3389/fpsyg.2019.02353)
Supplement: Supplementary file 1 [file Table_1.DOCX]

# Web Appendix: Getting Everyone Onboard: Framing Collective Goal Progress Broadens Participation in Collective Marketing Campaigns

## Sample Description Participants Viewed, Accumulating Condition with Two Stamps, Study 1:

Imagine that your friend recently moved away from the city where you live. Before they left, they gave you their partially completed reward card from a local cafe, which is shown below. This cafe is committed to decreasing its carbon footprint and reducing the amount of waste it produces. They have started a Waste Reduction Drive to eliminate 500 pounds of waste from landfills, the equivalent of around 10,000 disposable cups. You can participate in the project and help the cafe reach its goal by bringing your own mug to the cafe 10 times (helping eliminate around ½ pound of waste). Once you complete the reward card (bringing your own mug 10 times), you receive one free beverage as a reward. The reward card from your friend already has credit for bringing a mug two times. Green Café tracks participation in the Waste Reduction Drive in real time. The Drive has saved 8,500 cups from the landfill.

## Measurement items

| **Measurement items** |
| --- |
| Willingness to use a reward card  How likely is it that you would visit Green Café and use the reward card (1=*Definitely would not* to 7=*Definitely would*)? |
| Is Green Café’s goal to decrease waste desirable? (1 = *Strongly disagree* to 7 = *Strongly agree*)  Perceived impact of participation (*a* = .90)  To what extent does getting one stamp make you feel that you are making progress toward getting one free drink (1=*Not at all* to 7=*Very much*)?  How much progress would be using your own mug make toward the Green Café Waste Reduction Drive (1=*Not at all* to 7=*Extremely*)?  How big would your contribution be toward the Green Café Waste Reduction Drive if you purchased a drink using your own mug (1=*Not at all* to 7=*Extremely*)?  How substantial would your contribution be toward the Green Café Waste Reduction Drive if you purchased a drink using your own mug (1=*Not at all* to 7=*Extremely*)?  The participation of each additional person will have a significant effect on the likelihood of a successful drive (1=*Strongly disagree* to 7=*Strongly agree*).​  My participation will have a significant effect on the likelihood of a successful drive (1=*Strongly disagree* to 7=*Strongly agree*).​ |
| Feelings of helping one’s own community  To what extent does getting one stamp make you feel that you are helping your community (1=*Not at all* to 7=*Very much*)? |
| Willingness to bring one’s own mug  How likely is it that you would bring your own mug to Green Café in order to earn a stamp (1=*Definitely would not* to 7=*Definitely would*)? |
| Brand liking (*a* = .92)  I like the brand Green Café (1=*Strongly disagree* to 7=*Strongly agree*).  I admire the brand Green Café (1=*Strongly disagree* to 7=*Strongly agree*).  I feel like Green Café fits in my life (1=*Strongly disagree* to 7=*Strongly agree*). |
| Feelings of helping the environment  To what extent does getting one stamp make you feel that you are helping the environment (1=*Not at all* to 7=*Very much*)?  Evaluation of success for the goal (*a* = .76 in Study 1, *a* = .80 in Study 2)  To what extent do you trust Green Café to efficiently reduce its waste (1=*Not at all* to 7=*Very much*)?  To what extent does the progress of the Green Café Waste Reduction Drive thus far seem satisfying (1=*Not at all* to 7=*Extremely*)?  How likely is it that Green Cafe will reach its goal of eliminating 10,000 cups’ worth of waste (1=*Not likely* to 7=*Certainly*)? |
|  |

## Supplementary Tables


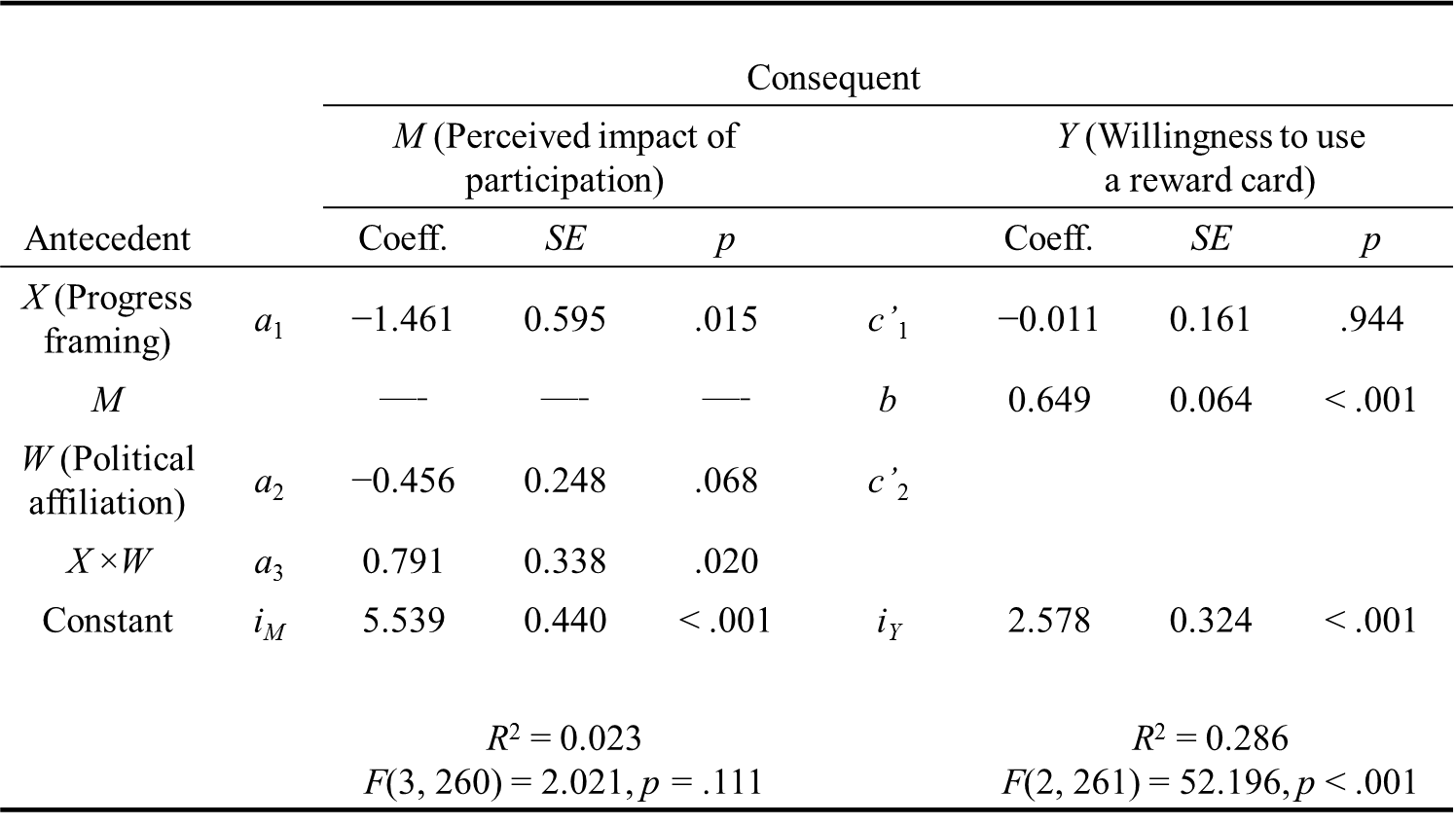


**Web Supplementary Table 1.** Perceived impact of one’s participation mediates the effects of progress framing on intention to participate in the waste reduction drive for Republicans, but not for Democrats.


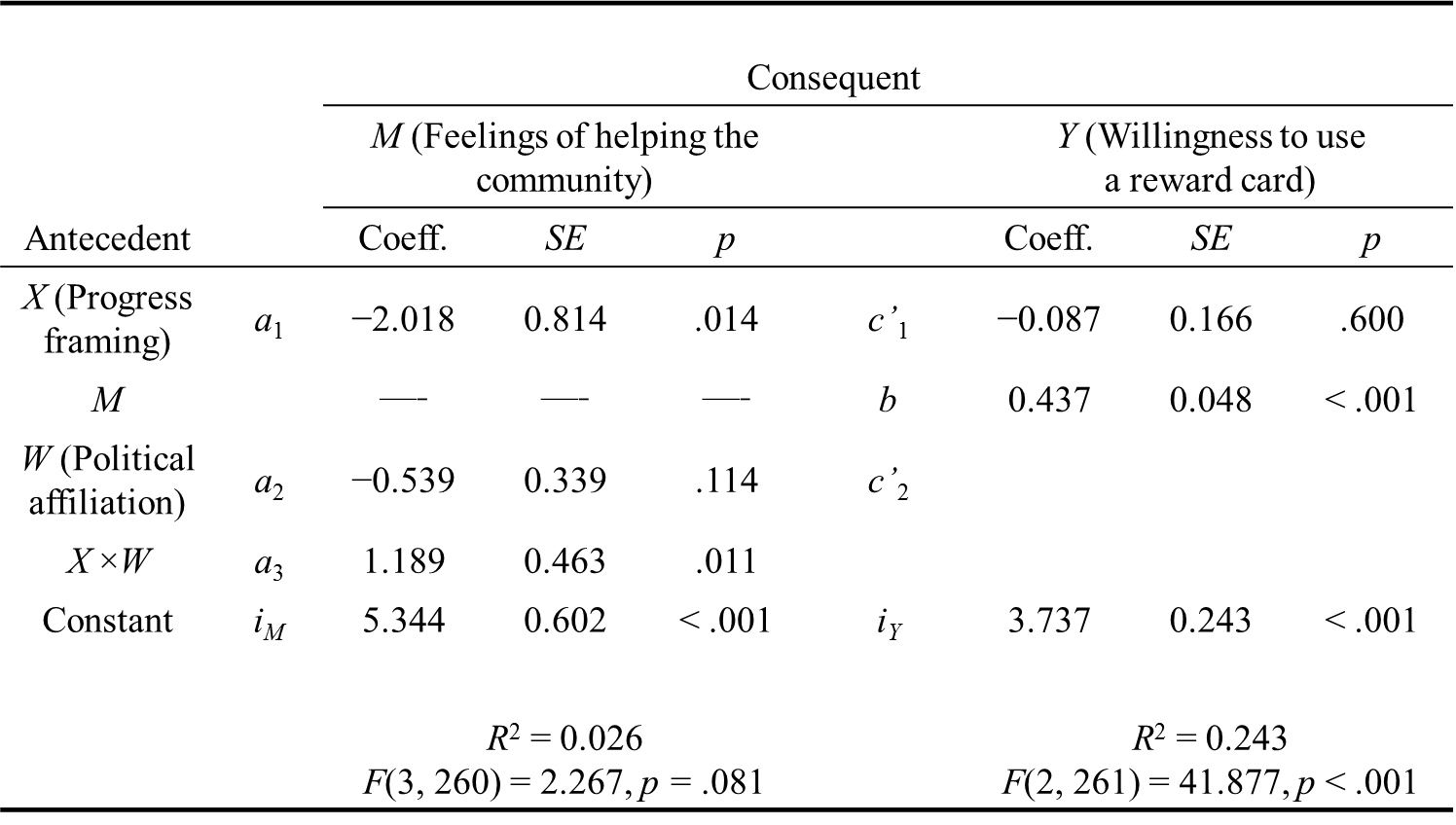


**Web Supplementary Table 2.** Feelings of helping the community mediate the effects of progress framing on intention to participate in the waste reduction drive for Republicans, but not for Democrats.
